# Supplementary material for: The feasibility of a telephone-based weight loss intervention in rural Ohio: A pilot study
Source: PLoS One. 2023 Mar 16;18(3):e0282719. doi: 10.1371/journal.pone.0282719 (PMC10019683; doi:10.1371/journal.pone.0282719)
Supplement: S1 Table — (DOCX) [file pone.0282719.s001.docx]

S1 Table. Study materials and supplementary resources by study group

|  | Telephone-based Health Counseling | Health Education |
| --- | --- | --- |
| Materials provided  at baseline | - A lifestyle modification manual - Exercise manual and online videos - Fitbit tracker - Weight scale - Self-log | - Education brochure (AICR guideline for physical activity and diet) - Exercise manual and online videos - Self-log |
| Other components provided | 15 weekly telephone sessions   - Health coach - provided education & counseling sessions - Weekly lifestyle education lessons - Tailored dietary modification, aerobic & resistance exercise recommendations - Individualized behavioral counseling - self-regulation strategies, social support & managing barriers - Group messages | At the end of the study   - A lifestyle modification manual - Fitbit tracker |
