# Supplementary material for: The feasibility of a telephone-based weight loss intervention in rural Ohio: A pilot study
Source: PLoS One. 2023 Mar 16;18(3):e0282719. doi: 10.1371/journal.pone.0282719 (PMC10019683; doi:10.1371/journal.pone.0282719)
Supplement: S2 Table — (DOCX) [file pone.0282719.s002.docx]

S2 Table. Telephone-based Health Counseling Intervention Components, Behavior Change Techniques (BCT), and Mechanisms of Action (MoA)

| **Weekly Telephone-based Health Counseling Sessions** | | **BCT [MoA]** |
| --- | --- | --- |
| **All sessions - opening/weekly review** | |  |
| Health coach discusses change in body weight; reviews EX+D logs; reviews and sets new weekly goals according to individual progress; prompts participant to identify barriers to meeting goals and ways to overcome them; asks participant to self-monitor using EX+D logs/Fitbit and record body weight; provide reinforcement and support participant as they meet intervention goals | | 1.1 [In, Go], 1.2 [BaCa, BR], 1.3 [Go, Mo], 1.4 [BC],1.5 [Go], 1.7 [Go], 2.2 [Mo, FP], 2.3 [BR, FP], 2.4, 2.7 [FP], 9.1 [Attb, GAB], 10.4 [Re, SI] |
| **All sessions - closing/weekly reflection** | |  |
| Health coach prompts reflection on reasons for wanting to be physically active and eat healthy; reviews changes made to behavioral goals, successes and barriers, and identifies level of social support in place to manage lapses; prompts participant to record weekly plan including daily macronutrient goal range, exercise goal, and step goal | | 2.3 [BR,FP], 3.1 [SI], 15.3 [BaCa] |
| **Supplementary strategies - used as needed** | |  |
| Health coach provides opportunity for consultation with licensed dietitian for review of food logs and feedback | | 2.2 [Mo, FP], 9.1 [Attb, GAB] |
| Health coach reviews home-based exercise manual including coaching of proper strength training form  Health coach encourages social support through peer-initiated group messaging; prompts sharing of progress and challenges; provides verbal reinforcement and affirmations of behavioral capabilities | | 4.1 [Kn, Sk, BaCa], 6.1 [BaCa, SLI], 8.1 [Sk, BaCa] |
|  |  | 2.2 [Mo, FP], 15.1 [BaCa] |
| **Week 1** | Health coach discusses health consequences of EX+D and weight loss on cancer risk, overall health benefits, and social and emotional implications | 5.1 [Kn, BaCo, In, Attb, Psv], 5.6 [BaCo], 9.1 [Attb, GAB] |
|  | Health coach advises on strength training principles, safety, and proper form; introduces home-based exercise packet; participant is asked to practice exercises at home | 4.1 [Kn, Sk, BaCa], 6.1 [BaCa, SLI], 8.1 [Sk,BaCa], 8.3 [BC], 12.6 |
|  | Health coach and participant agree on 15-week individual goal of ~7% weight loss via gradual lifestyle change | 1.1 [In, Go], 1.3 [Go, Mo] |
|  | Health coach introduces dietary self-monitoring tool; explains use; instructs participant to log daily; prompts participant to submit logs weekly for feedback | 2.2 [Mo, FP], 2.3 [FP], 4.1 [Kn, Sk, BaCa], 6.1 [BaCa, SLI] |
|  | Health coach introduces physical activity self-monitoring device; explains use; instructs participant to log steps and active minutes | 2.3 [BR, FP], 4.1 [Kn, Sk, BaCa], 6.1 [BaCa, SLI] |
| **Week 2** | Health coach introduces evidence-based dietary guidelines; explains how to find ingredients and nutrient information; explains calculation of calorie budget goals | 4.1 [Kn, Sk, BaCa], 6.1 [BaCa, SLI] |
|  | Health coach and participant agree on goals of ~150 min of moderate intensity physical activity and total body strength training 2x/week | 1.1 [In, Go] |
| **Week 3** | Health coach introduces the FITT principle for EX prescription, explains rating of perceived exertion and safe EX technique | 4.1 [Kn, Sk, BaCa], 6.1 [BaCa, SLI] |
|  | Health coach introduces and explains SMART goal-setting tool for EX+D and weight loss goals; prompts participant to create a SMART goal | 1.1[In, Go], 1.3  [BaCa, BR, Go, Mo], 4.1 [Kn, Sk, BaCa], 6.1 [BaCa, SLI], 8.1 [Sk, BaCa] |
|  | Health coach introduces IDEA barrier problem-solving tool; demonstrates use of tool by prompting participant to identify barrier to EX+D, brainstorm potential solutions, prompt substitutions for behaviors, evaluate the options and results | 1.2 [BaCa, BR], 1.4 [BC], 8.2 [BR] |
| **Week 4** | Health coach introduces evidence-based dietary best-practices and food groups including sample meal options for fruits and vegetables | 4.1 [Kn, Sk, BaCa], 6.1 [BaCa, SLI] |
| **Week 5** | Health coach facilitates discussion of barriers to EX; prompts participant to record new activities they would like to include in weekly plan; encourages writing action plan including the activity, time and planned active minutes | 1.1 [In, Go], 1.2 [BaCa, BR], 1.4 [BC], 1.6 [Go, FP] |
| **Week 6** | Health coach introduces benefits of dietary protein intake; advises on consuming best sources; demonstrates and prompts calculation of individual protein goals | 1.1 [In, Go], 1.4 [BC], 4.1 [Kn, Sk, BaCa], 5.1 [Kn, BaCo, In, Attb, Psv], 6.1 [BaCa, SLI] |
|  | Health coach prompts planning and self-monitoring of dietary protein; provides feedback on weekly protein intake; prompts high protein substitutions; recommends protein intake at each meal | 2.3 [BR, FP], 4.1 [Kn, Sk, BaCa], 6.1 [BaCa, SLI], 8.1-8.3 [Sk, BaCa, BR, BC] |
|  | Health coach explains how to identify dietary fat; advises on consuming best sources; prompts planning for managing dietary fat; provides feedback on weekly fat intake; prompts substitutions | 2.3 [BR, FP], 4.1 [Kn, Sk, BaCa], 6.1 [BaCa, SLI], 8.2 [BR] |
| **Week 7** | Health coach introduces causes and effects of stress, benefits of stress management including physical and emotional consequences; prompts noticing of reaction to stress and how to manage; demonstrates relaxation techniques; participant practices breathing exercises | 1.2 [BaCa, BR], 4.1 [Kn, Sk, BaCa], 5.1 [Kn, BaCo, In, Attb, Psv], 5.4, 5.6 [BaCo], 6.1 [BaCa, SLI], 8.1 [Sk. BaCa], 11.2 [Em, BR] |
| **Week 8** | Health coach explains portion control; provides examples of common units of measurement; prompts measuring food practice; facilitates discussion on mindful eating; prompts participant to record awareness of eating in various situations | 4.1 [Kn, Sk, BaCa], 4.3, 6.1 [BaCa, SLI], 8.1 [Sk. BaCa] |
|  | Health coach explains effects of added sugar on health; advises on managing sugar intake | 4.1 [Kn, Sk, BaCa], 5.1 [Kn, BaCo, In, Attb, Psv] |
|  | Health coach introduces the benefits of fiber and its effects on health; advises on increasing fiber intake | 4.1 [Kn, Sk, BaCa], 5.1 [Kn, BaCo, In, Attb, Psv] |
| **Week 9** | Health coach facilitates discussion of behavioral strategies to remain active; prompts participant to create commitment document including “I will” statements; encourages participant to use commitment document as a reminder | 1.2 [BaCa, BR], 1.4 [BC], 1.8, 1.9, 7.1 [MADP, ECR, BC], 8.3 [BC] |
| **Week 10** | Health coach facilitates discussion of strategies of mindful eating while dining out; prompts participant to reflect on and record barriers to healthy eating in these situations; prompts participant to record ways to change environment for mindful eating at home, work and in between (car, travel, etc.) and encourage implementation of these strategies | 1.2 [BaCa, BR], 8.1-8.3 [Sk, BaCa, BR, BC], 12.1 [ECR, BC] |
| **Week 11** | Health coach reviews weight loss goal; provides feedback on current progress; introduces accountability planning for weight loss maintenance; prompts participant to identify supportive family/friends and create an accountability plan to enhance weight loss maintenance | 1.7 [Go], 2.7 [FP], 3.1 [SI], 4.1 [Kn, Sk, BaCa], 6.1 [BaCa, SLI], 15.1 [BaCa] |
| **Week 12** | Health coach facilitates IDEA barrier problem solving using practice scenario; prompts brainstorming of appealing healthy substitutions for favorite foods | 1.2 [BaCa, BR], 1.4 [BC], 6.1 [BaCa, SLI], 8.1-8.2 [Sk, BaCa, BR] |
| **Week 13** | Health coach explains the benefit of sleep and sleep hygiene including physical and emotional consequences; revisits stress management techniques; prompts discussion of common reactions to stress to take control of cognitions | 1.2 [BaCa, BR], 4.1 [Kn, Sk, BaCa], 5.1 [Kn, BaCo, In, Attb, Psv], 5.6 [BaCo], 6.1 [BaCa, SLI] |
| **Week 14** | Health coach facilitates discussion of stress eating, feelings, and cravings; illustrates the differences between physical, emotional and sensory hunger; cues participant to identify hunger; suggests alternative explanations for appetite; advises participant to eliminate food triggers in home; prompts participant to create a relapse prevention plan for cravings | 1.2 [BaCa, BR], 1.4 [BC], 4.1 [Kn, Sk, BaCa], 4.3, 6.1 [BaCa, SLI], 11.2 [Em, BR], 12.1 [ECR, BC] |
| **Week 15** | Health coach prompts participant to record changes in EX+D behaviors and weight loss at the end of the program | 1.5 [Go], 1.6 [Go, FP], 1.7 [Go] |
|  | Health coach discusses changes in the last 15-weeks and facilitates review of the self-regulation “toolbox for success” including self-monitoring, goal setting, problem solving, positive self-talk, celebrating achievements, etc. | 1.4 [BC], 8.3 [BC], 13.1 [SI], 15.1 [BaCa], 15.3 [BaCa], 15.4 [BaCa, Mo] |
| *BCT:* Behavior Change Technique*. MoA:* Mechanism of Action*. EX+D:* Exercise and Diet*. FITT:* Frequency, Intensity, Type, Time. *SMART:* Specific, Measurable, Achievable/Appealing, Realistic, Time-sensitive. *IDEA:* Identify, Develop, Evaluate, Analyze. *Kn:* Knowledge*. Sk:* Skill*. BaCa:* Beliefs about capabilities*. BaCo:* Beliefs about consequences*. Re:* Reinforcement*. In:* Intention*. Go:* Goals*. MADP:* Memory, attention & decision processes*. ECR:* Environmental context & resources*. Si:* Social influences*. Em:* Emotion*. BR:* Behavioral regulation*. No:* Norms*. SN:* Subjective norms*. Attb:* Attitude towards the behavior*. Mo:* Motivation*. Si*: Self-image*. FP:* Feedback processes*. SLI:* Social learning/imitation*. BC:* Behavioral cueing*. GAB:* General attitudes/beliefs*. Psv:* Perceived susceptibility/vulnerability*.* Conclusive links between BCTs and MoAs *s*ourced from Carey et al., 2019; https://theoryandtechniquetool.humanbehaviourchange.org/tool. | | |
